# Screening and epitope characterization of Nidogen‐2‐specific nanobodies

**DOI:** 10.1002/2211-5463.70219

**Published:** 2026-03-04

**Authors:** Jianchuan Wen, Qianqian Cui, Zhongyun Lan, Yingjun Wang, Shuaiying Zhao, Wenxuan Feng, Yunfeng Liu, Qiting Huang, Dongna Zhang, Jianfeng Xu

**Affiliations:** ^1^ College of Food Science and Technology Shanghai Ocean University China; ^2^ Shanghai Institute of Materia Medica Chinese Academy of Sciences China; ^3^ School of Pharmacy Nanjing University of Chinese Medicine China

**Keywords:** nanobody, Nidogen‐2 (NID2), phage display, Sandwich ELISA

## Abstract

NID2 is a key component of the BM and plays an important role in ECM organization and tumor‐associated microenvironmental remodeling. In this study, full‐length recombinant NID2 was used as an antigen to immunize camels, and a nanobody phage display library was constructed from peripheral blood lymphocytes. Using phage display‐based screening, nanobodies recognizing distinct epitopes within the NID2 G1G2 and rod–G3 domains were isolated and characterized. Epitope specificity and competition were further analyzed by BLI, allowing the identification of nonoverlapping nanobody pairs. Based on these results, two high‐affinity nanobodies were selected to establish a nanobody‐based sandwich ELISA for NID2 detection. This assay enabled the detection of recombinant NID2 at concentrations down to 10 ng·mL^−1^ in human serum. Although the analytical sensitivity is lower than that of some commercial antibody‐based ELISA kits, the nanobody‐based format offers advantages in terms of recombinant production, scalability, and assay design flexibility. Collectively, this study provides a panel of domain‐selective nanobodies against NID2 and establishes a nanobody‐based sandwich ELISA as a methodological platform for future investigations of serum NID2 in diagnostic and translational research contexts.

## Graphical abstract

Camel immunization and phage display were employed to generate high‐affinity VHH nanobodies against Nidogen‐2. After library construction, biopanning, ELISA screening, sequencing, and recombinant expression, selected nanobodies were purified and characterized, leading to the preliminary exploration of a nanobody‐based sandwich ELISA for specific Nidogen‐2 detection.

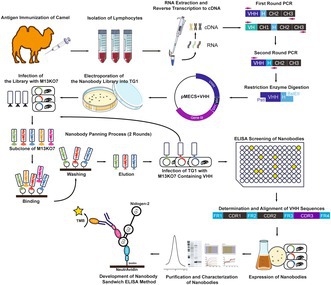

**Abbreviations**
BLI: biolayer interferometry
BM: basement membrane
BSA: bovine serum albumin
CDR3: complementarity‐determining region 3
ECM: extracellular matrix
ELISA: enzyme‐linked immunosorbent assay
FL: full‐length
HEK: Human embryonic kidney
HQC: high‐quality control
HRP: horseradish peroxidase
LGR4: leucine‐rich repeat containing G protein‐coupled receptor 4
LQC: low‐quality control
NB: nanobody
NCBI: National Center for Biotechnology Information
NID1: Nidogen‐1
NID2: Nidogen‐2
OD: optical density
PBS: phosphate‐buffered saline
PTM: post‐translational modification
RSPO1: R‐spondin 1
RSPO2: R‐spondin 2
SDS/PAGE: sodium dodecyl sulfate/polyacrylamide gel electrophoresis
SEC: size‐exclusion chromatography
TMB: 3,3′,5,5′‐Tetramethylbenzidine
VHH: variable domains of camelid heavy‐chain antibodies

## Introduction

**Fig. 1:**
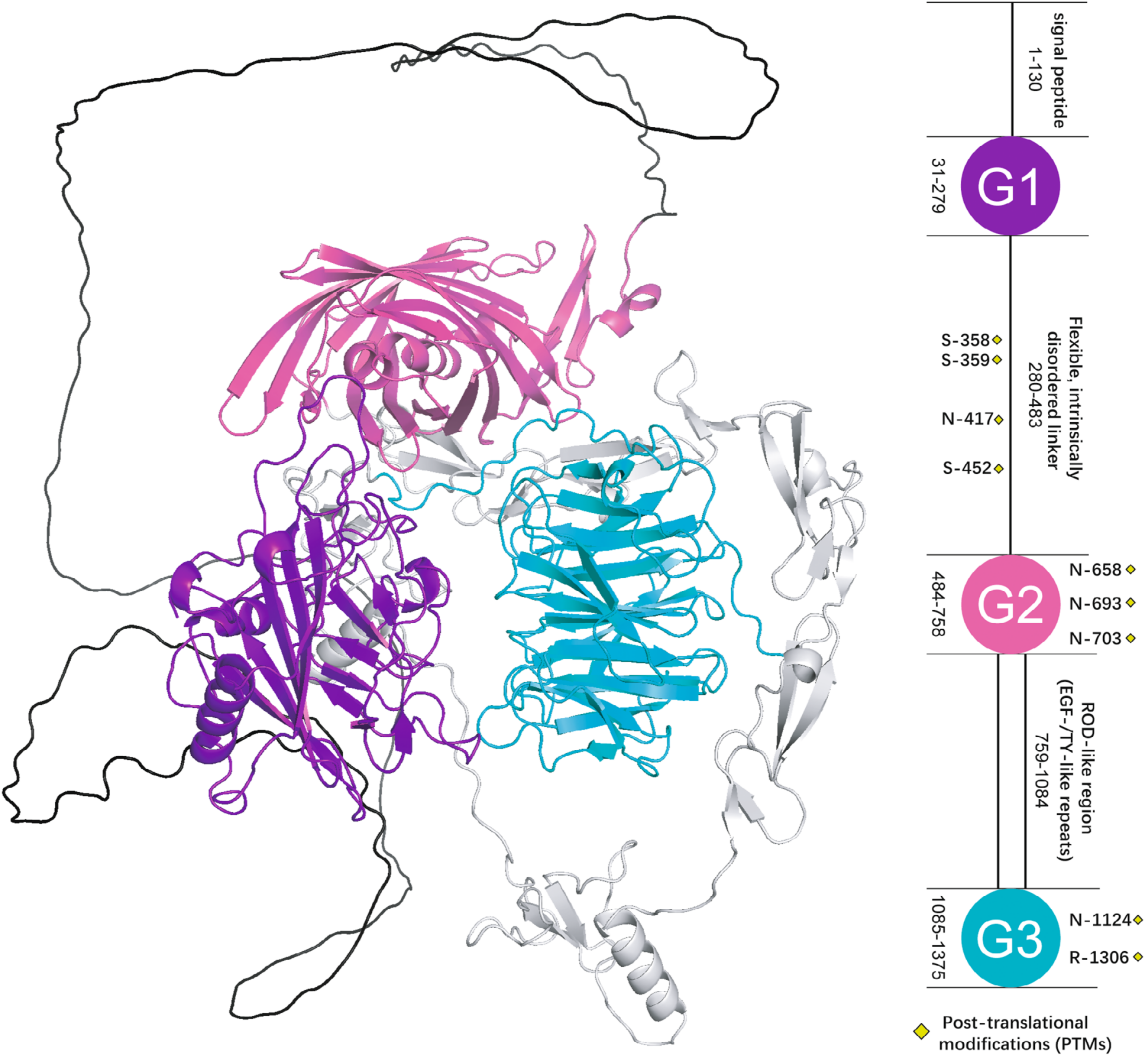
Domain organization of human NID2. The schematic illustration is based on protein structure prediction using alphafold3, with domains colored as G1 (purple), G2 (magenta), and G3 (cyan). The illustration highlights the flexible intrinsically disordered linker connecting the G1 and G2 domains and the rigid rod‐like region composed of EGF‐like repeats linking the G2 and G3 domains. Domain boundaries, residue ranges, and annotated post‐translational modification (PTM)–prone residues are indicated to facilitate interpretation of domain‐specific nanobody binding.

Nidogen‐2 (NID2) is an important glycoprotein localized in the extracellular matrix (ECM) with a molecular weight of approximately 200 kDa. It is widely distributed in the basement membrane (BM), with a length of approximately 40–50 nm and three globular domains (G1, G2, and G3) [1] (Fig. 1). Domains G1 and G2 are connected by a flexible linker, while domains G2 and G3 are linked by a rigid rod‐like region [2]. The G1G2 domain has been reported to be involved in PKCα‐associated intracellular signaling pathways and plays an important role in cell signaling regulation [3]. The rod–G3 domain can connect collagen IV and laminin and is involved in the assembly and stabilization of the BM [4]. Abnormal expression of NID2 is associated with tumors and tissue fibrosis diseases [5]. Therefore, the G1G2 and rod–G3 domains of NID2 represent important targets for tool development and mechanistic studies related to these diseases.

NID2 is a promising diagnostic biomarker for multiple cancers, including ovarian cancer [6], colon cancer [7], gastric cancer [8, 9], and nonsmall cell lung cancer [10]. In addition to tissue‐level alterations, circulating NID2 or NID2‐derived forms in serum or plasma have been proposed as potential noninvasive diagnostic indicators in several malignancies. However, robust and scalable detection methods suitable for serum‐based NID2 analysis remain limited.

Nanobodies are the isolated variable domains of camelid heavy‐chain antibodies (VHH). These ~15 kDa domains are naturally evolved to bind antigen in the absence of a paired light chain and are generally soluble and simple to express recombinantly [11]. Owing to their extended complementarity‐determining region 3 (CDR3), nanobodies can access cryptic or recessed epitopes that are often inaccessible to conventional antibodies [12]. Moreover, their high structural stability enables them to maintain functionality under harsh conditions, making nanobodies particularly suitable for the development of sensitive and specific immunoassays. In recent years, nanobody‐based enzyme‐linked immunosorbent assay (ELISA) platforms have been successfully applied to the detection of viral antigens and disease‐related antibodies, highlighting their potential in diagnostic applications [13, 14].

In this study, nanobodies targeting the G1G2 and rod–G3 domains of NID2 were screened using phage display technology [15]. By characterizing their epitope specificity and binding properties, we aimed to generate domain‐selective nanobody reagents and to explore their application in the development of a nanobody‐based sandwich ELISA for NID2 detection. This work provides molecular tools for investigating NID2 biology and establishes a methodological foundation for future diagnostic and translational studies involving NID2‐related diseases [8, 12].

## Materials and methods

### Main materials

#### Main reagents and instruments

- UE PCR Product Purification Kit (cat. no. UE‐PCR‐500) and UE DNA Gel Extraction Kit (cat. no. UE‐GX‐500) were purchased from Suzhou Youyi Landy Biotechnology Co., Ltd., Suzhou, Jiangsu, China.
- Biotin Labeling Kit for Avi‐tag Protein with BirA (cat. no. P0630M) was purchased from BeyoTime Co., Ltd., Shanghai, China.
- DNA polymerase (cat. no. P101‐03), ClonExpress II One Step Cloning Kit (cat. no. C112‐01), T4 DNA ligase (cat. no. C301‐01), and RNA extraction kit (cat. no. RC102‐01) were purchased from Nanjing Vazyme Biotech Co., Ltd., Nanjing, Jiangsu, China;
- Molecular interaction instrument (OCTET Red96e) was purchased from Forte Biosciences, Inc., Dallas, TX, USA.
- Streptavidin (SA) biosensors (cat. no. 18‐5019) were purchased from Sartorius AG, Göttingen, Germany;
- NeutrAvidin (cat. no. 31001) and HRP‐conjugated HA Tag mouse monoclonal antibody (cat. no. 26183‐HRP) were purchased from Thermo Fisher Scientific, Waltham, MA, USA.
- A Spark^®^ multimode microplate reader (Tecan, Männedorf, Switzerland) was used for absorbance measurements.
- Superdex™ 200 Increase 10/300 GL and HiLoad™ Superdex™ 75 pg size‐exclusion chromatography (SEC) columns, as well as the ÄKTA™ pure chromatography system, were purchased from Cytiva (Shanghai) Co., Ltd.
- M2 anti‐FLAG affinity resin and Ni‐NTA affinity resin were purchased from Nanjing GenScript Biotech Co., Ltd.

#### Main strains and vectors

The bacterial strains, cells, and plasmids used in this study are listed in Table 1.

**Table 1:** The *E. coli* strains, cells, and plasmids used in this study.

*E. coli* strains	Source
TG1	Agilent
WK6	NEB
DH5α	NEB
Plasmids	Source
pCMV‐NID2‐FL‐Flag	SinoBiological
pCMV‐NID2‐G1G2‐Flag	This study
pCMV‐NID2‐FL‐AVI‐Flag	This study
Pet28a‐HIS	This study
pMECS‐HA‐HIS	This study
Cell lines	Source
HEK‐293F	SinoBiological

##### Cell line authentication and mycoplasma testing

The Human embryonic kidney 293F (HEK‐293F) cell line used in this study was purchased from Sino Biological Inc., Beijing, China. The cell line was authenticated by the supplier within the past 3 years using short tandem repeat (STR) profiling in accordance with standard cell line authentication procedures and was confirmed to be free of cross‐contamination prior to use. All cell cultures were routinely tested for mycoplasma contamination using a commercial detection kit, and all experiments were performed with mycoplasma‐free cells.

### Construction, expression, and purification of NID2‐related antigen proteins

#### Construction of eukaryotic expression vectors for NID2‐FL‐Avi and NID2‐G1G2

The gene sequence of human NID2 was obtained from the National Center for Biotechnology Information (NCBI) database. To maximize the number of nanobodies binding to NID2 screened by phage display, an Avi tag (GLNDIFEAQKIEWHE) was fused to the C terminus of NID2, and the pCMV‐NID2‐FL‐Avi‐Flag plasmid was constructed. To distinguish nanobodies targeting different structural domains of NID2, the amino acid fragment corresponding to the G1G2 domain was inserted into pCMV. The full‐length NID2 gene sequence (amino acids 1–1375) served as the template for amplification, while pCMV‐Flag was used as the vector backbone. Homologous recombination PCR amplification was performed to generate NID2‐FL‐Avi and NID2‐G1G2 fragments (Table 2), which were then ligated into the pCMV‐Flag vector via Exnase II‐mediated recombination between the homologous arms of the insert and the vector.

**Table 2:** Primers used for vector construction.

Primer name	Primer sequence
NID2‐F	5′‐CCACCAAGCTTGGTACCATGGAGGGGGACCGGGTG‐3′
NID2‐R	5′‐CGTCATCCTTGTAATCAGAGCCTCCACCCCCCTTTCTTCCTGTT‐3′
pCMV‐NID2‐F	5′‐GGGTGGAGGCTCTGATTACAAGGATGACGACGA‐3′
NID2‐G1G2‐F	5′‐TAGGGGCCGCCACCAAGCTTGGTACCATGGAGGGGGACCGGGT‐3′
NID2‐G1G2‐R	5′‐CAGAGCCTCCACCCCCAGTGGGGTCTGAATCTTC‐3′
pCMV‐G1G2‐F	5′‐GGGGGTGGAGGCTCTGATTACAAGGATGACGACGATAAG‐3′
pCMV‐G1G2‐R	5′‐ACCCGGTCCCCCTCCATGGTACCAAGCTTGGTGGCGGCCCCTA‐3′

#### Expression and purification of NID2‐FL, NID2‐FL‐Avi, and NID2‐G1G2

The endotoxin‐removed pCMV‐NID2‐FL‐Flag, pCMV‐NID2‐FL‐Avi‐Flag, and pCMV‐NID2‐G1G2‐Flag plasmids were transiently transfected into HEK‐293F cells (2.3 × 10^6^ cells·mL^−1^). After transfection, the cells were cultured at 37 °C with 8% CO_2_ for 12 h, then 10 mmol·L^−1^ sodium butyrate was added, and the temperature was reduced to 30 °C for further culture for 72 h. After collecting the supernatant, an equilibration buffer (20 mmol·L^−1^ HEPES, pH 7.4; 150 mmol·L^−1^ NaCl; 5 mmol·L^−1^ EDTA; 1× penicillin–streptomycin) was added, mixed well, and filtered through a 0.22 μm filter membrane. The filtered supernatant was slowly passed through the M2 anti‐FLAG affinity resin at 4 °C to allow the target protein to bind to the resin. Impurities were eluted with a wash buffer (20 mmol·L^−1^ HEPES, pH 7.4; 150 mmol·L^−1^ NaCl), and then the protein was eluted with an elution buffer (20 mmol·L^−1^ HEPES, pH 7.4; 150 mmol·L^−1^ NaCl; 0.25 mg·mL^−1^ FLAG peptide). The eluted product was concentrated to 500 μL using a 50 kDa ultrafiltration tube and further purified by protein separation using a Superdex™ 200 Increase 10/300 GL Increase chromatography column. The molecular weight and purity of NID2‐FL, NID2‐FL‐Avi, and NID2‐G1G2 proteins were verified by SDS/PAGE. *In vitro* biotinylation of NID2‐FL‐Avi was performed using a BirA‐based biotinylation system at 30 °C for 40 min under the conditions summarized in Table 3. Detection of biotinylated NID2‐FL‐Avi was performed by streptavidin–horseradish peroxidase (HRP) blotting. Protein samples were separated by SDS/PAGE and transferred onto PVDF membranes. After blocking, membranes were incubated with HRP‐conjugated streptavidin to detect biotinylated protein species. Signals were visualized using enhanced chemiluminescence (ECL) reagents and recorded by exposure‐based imaging.

**Table 3:** Biotinylation system for NID2‐FL‐Avi.

Reagent	Volume (μL)
BirA	22
NID2‐FL‐Avi (1 mg·mL^−1^)	1000
10× Biotin‐L‐Buffer‐A	128
10× Biotin‐L‐Buffer‐B	128
Total	1278

### Screening and characterization of anti‐NID2 Nanobodies

Unless otherwise stated, all ELISA and biolayer interferometry (BLI)‐based assays used for the screening and characterization of anti‐NID2 nanobodies were performed using detergent‐free buffer systems without Tween‐20. This strategy was adopted to minimize potential effects of surfactants on the conformation of NID2 and on the accessibility of conformational epitopes.

For ELISA assays in this section, phosphate‐buffered saline (PBS, pH 7.4) supplemented with 2% (w/v) bovine serum albumin (BSA) was used as the blocking buffer, whereas PBS supplemented with 0.2% (w/v) BSA was used as the wash buffer as well as for sample and antibody dilution. All ELISA incubations were performed at 22 °C on a horizontal shaker under gentle agitation.

For BLI measurements, assays were conducted in Octet buffer consisting of 20 mmol·L^−1^ HEPES (pH 7.4), 150 mmol·L^−1^ NaCl, and 0.2% (w/v) BSA at 22 °C, using the default shaking speed recommended by the instrument manufacturer.

#### Animals and ethical statement

All animal procedures were conducted in accordance with institutional guidelines for animal care and use and complied with the ARRIVE 2.0 guidelines (Essential 10). The study protocol was approved under the license SCXK (沪) 2015–0004 issued to the Shanghai Institute of Materia Medica, Chinese Academy of Sciences.

##### Animal housing and husbandry

Camels used in this study were housed in a licensed, specialized breeding facility. Animals were maintained under natural photoperiod conditions consistent with the local light–dark cycle in Shanghai. Camels had *ad libitum* access to clean drinking water sourced from potable tap water meeting human consumption standards and were fed a standard diet consisting of hay and grain. Housing conditions were routinely monitored to ensure appropriate space, ventilation, and environmental hygiene.

##### Animal characteristics

Healthy adult camels of both sexes were used in this study. All animals were young‐ to middle‐aged adults, with an estimated age range of 4–8 years, and were clinically assessed to be in good health prior to experimental procedures.

##### Measures to minimize animal suffering

All experimental procedures were performed by trained personnel to minimize stress and discomfort. During immunization, blood collection, and injection procedures, animals were gently restrained, and handling time was kept as short as possible. All procedures were carried out using sterile equipment and appropriate techniques to reduce pain and tissue injury. Animals were closely monitored during and after experimental procedures, and no unexpected adverse effects were observed.

#### Camel immunization

The purified NID2‐FL protein was aliquoted at a concentration of 2 mg·mL^−1^, with 1 mL per tube, totaling seven tubes. To preserve the protein, 15% glycerol was added to each aliquot, which was then snap‐frozen in liquid nitrogen and stored at −80 °C. Camels were immunized once a week; before each immunization, 1 mL of adjuvant was mixed with the protein, and the mixture was injected. The immunization lasted for 7 weeks.

#### cDNA construction

After the completion of immunization, 90 mL of peripheral blood was collected from the area near the camel's lymph nodes, and 90 mL of normal saline was added to dilute the peripheral blood. The diluted blood was then divided into 12 50‐mL centrifuge tubes, with 15 mL per tube, and 10 mL of lymphocyte separation medium was added to each tube. After centrifugation, the blood was separated into three layers, and the middle lymphocyte layer was carefully extracted. Subsequently, mRNA was extracted from the lymphocytes using an RNA extraction kit. The extracted mRNA was verified by 2% agarose gel electrophoresis, and the results showed that distinct bands were formed for both 28S and 18S rRNA. The qualified mRNA was then reverse‐transcribed into cDNA and stored frozen in a −20°C refrigerator.

#### Construction of an anti‐NID2 Nanobody library

The cDNA was subjected to two rounds of nested PCR (Table 4) to obtain nanobody sequence fragments. The fragments were digested with restriction enzymes (restriction sites: BamHI, XbaI) together with the pMECS vector. After ligation of the vector and fragments, the mixture was electroporated into TG1 competent cells. The electroporated TG1 competent cells were resuscitated and spread on 2YT‐AMP‐GLU plates (2YT medium plates containing 0.1 g·L^−1^ ampicillin and 2 g·L^−1^ glucose), followed by static culture in a 37 °C incubator overnight for 12 h. The bacterial lawn was scraped off the plates and collected into 50‐mL centrifuge tubes, totaling 30 mL. A 10 μL aliquot of this bacterial suspension was diluted with LB medium to 3 mL, and its optical density (OD) value was measured using a spectrophotometer. 7 mL of glycerol was added to the bacterial suspension to prepare a glycerol stock library, which was then quickly frozen in liquid nitrogen and stored in a −40 °C refrigerator.

**Table 4:** Primers used for two‐round nested PCR.

Primer name	Primer sequence
CALL‐F	5′‐GTCCTGGCTGCTCTTCTACAAGG‐3′
CALL‐R	5′‐GGTACGTGCTGTTGAACTGTTCC‐3′
VHH‐F	5′‐GATGTGCAGCTGCAGGAGTCTGGRGGAGG‐3′
VHH‐R	5′‐CTAGTGCGGCCGCTGGAGACGGTGACCTGGGT‐3′

#### Amplification of M13KO7 helper phage

A single colony of TG1 was inoculated into 2YT medium containing 2 g·L^−1^ glucose and 0.1 g·L^−1^ ampicillin and cultured at 37 °C with shaking at 220 rpm until the OD600 was < 0.05. M13KO7 stock solution was then added, and the culture was incubated statically at 37 °C for 30 min. After centrifugation, the supernatant was discarded, and the pellet was resuspended in 500 mL of 2YT medium containing 0.1 g·L^−1^ ampicillin, followed by culture at 37 °C with shaking at 200 rpm for 12 h. After multiple centrifugations, 0.25 volumes of PEG‐NaCl were added to the supernatant, and the mixture was placed on ice for 40 min. After another centrifugation, the pellet was resuspended in PBS, and undissolved precipitates were removed. The phage supernatant was diluted and spread on LB‐AMP medium, cultured at 37 °C for 12 h, then the titer was determined and the phage was stored at 4 °C.

#### Amplification and titer determination of the anti‐NID2 Nanobody library

The glycerol stock library was inoculated into 100 mL of 2YT medium containing 2 g·L^−1^ glucose and 0.1 g·L^−1^ ampicillin and cultured until the OD600 reached 0.4–0.5. M13KO7 phage was added, and the culture was incubated statically at 37 °C for 30 min, then centrifuged to discard the supernatant. After drying, the pellet was resuspended in 2YT medium and transferred to 500 mL of 2YT medium containing 0.1 g·L^−1^ ampicillin and 0.1 g·L^−1^ kanamycin, followed by culture at 37 °C with shaking at 220 rpm for 12 h. The supernatant was collected by centrifugation; 0.25 volumes of PEG‐NaCl were added, and the mixture was placed on ice for 30 min. After another centrifugation, the pellet was dried and resuspended in PBS. After multiple centrifugations until no precipitate remained, the phage solution was diluted and spread on plates to determine the phage titer.

#### Panning of the anti‐NID2 Nanobody library

NeutrAvidin stock solution was diluted to a concentration of 10 μg·mL^−1^, and 100 μL per well was added to a plate, followed by incubation at 4 °C overnight. The next day, the plate was washed 5 times with PBST, and 250 μL per well of 5% BSA blocking solution was added, followed by incubation at room temperature for 2 h. After discarding the blocking solution, the plate was washed five times with PBST, and blocking solution containing the antigen was added (no antigen was added to the control wells), followed by incubation at room temperature for 30 min. After washing, 100 μL per well of diluted phage solution was added, and the plate was incubated at room temperature for 2 h. Subsequently, 10 washes with PBST and 5 washes with PBS were performed, and trypsin was added for digestion at room temperature for 30 min. The trypsin was aspirated, AEBSF was added to terminate the reaction, and the screened phage library was diluted and spread on plates to determine the enrichment degree. The amplification steps of the sub‐library were the same as those for the first‐round library amplification, and finally, the enrichment degree was determined to meet the experimental requirements.

#### Screening and purification of anti‐NID2 Nanobodies

##### High‐throughput screening of anti‐NID2 nanobodies

The second‐round antibody library was diluted in PBS and plated onto 2YT–AMP–GLU agar plates. A total of 190 single colonies were picked and inoculated into two 96‐well deep‐well plates, each well containing 150 μL of 2YT medium supplemented with 0.1 g·L^−1^ ampicillin (one well per plate was kept as a blank control). Plates were incubated at 37 °C for 12 h. Subsequently, 15 μL of each culture was transferred into a fresh 96‐well plate containing 135 μL 2YT medium with 0.1 g·L^−1^ ampicillin and cultured at 37 °C with shaking at 220 rpm for 8 h. Protein expression was induced by adding 1 mm IPTG followed by incubation at 28 °C and 180 rpm for 12 h.

After induction, cultures were centrifuged, supernatants were discarded, and pellets were resuspended in lysis buffer. To release periplasmic nanobodies, plates were placed in a 37 °C water bath for 1 min and then rapidly frozen in liquid nitrogen for 30 s; this freeze–thaw cycle was repeated 10 times. Lysates were clarified by centrifugation, and supernatants were collected for ELISA. Nanobody binding to NID2 was assessed by measuring absorbance at 450 nm. Clones with OD450 values at least fivefold higher than the blank control were submitted to GENEWIZ (Suzhou, China) for sequencing.

##### Large‐scale expression and purification of anti‐NID2 nanobodies

Plasmids encoding nanobodies with distinct CDR3 sequences were transformed into WK6 competent cells. Following expansion in 800 mL culture, cells were harvested by centrifugation at 3565 ***g*** for 1 h at 4 °C. The cell pellet was resuspended in 20 mL lysis buffer (20 mmol·L^−1^ Tris–HCl, pH 8.0; 150 mmol·L^−1^ NaCl; 0.2 mmol·L^−1^ PMSF) and lysed by ultrasonication. The lysate was clarified by ultracentrifugation at 40 000 rpm for 1 h at 4 °C.

The supernatant was applied to Ni‐NTA affinity resin at 4 C to allow binding of the nanobody. After washing with 50 mL wash buffer (20 mmol·L^−1^ Tris–HCl, pH 8.0; 150 mmol·L^−1^ NaCl; 20 mmol·L^−1^ imidazole), the protein was eluted with 15 mL elution buffer (20 mmol·L^−1^ Tris–HCl, pH 8.0; 150 mmol·L^−1^ NaCl; 150 mmol·L^−1^ imidazole).

The eluate was concentrated to 5 mL using a 15‐kDa cutoff centrifugal concentrator (1164 ***g***, 4 C, 15 min·cycle^−1^) and further purified using a HiLoad™ Superdex 75 pg SEC column. The molecular weight and purity of the nanobody were assessed by SDS–PAGE.

##### Preliminary epitope identification of anti‐NID2 nanobodies

Purified nanobodies were subjected to ELISA using NID2‐FL and NID2‐G1G2 as coating antigens. Based on OD450 values measured using a microplate reader, nanobodies that preferentially recognized the G1G2 or G3 domains of NID2 were preliminarily identified.

#### Nanobody competition assay against different domains of NID2

BLI was employed to evaluate epitope competition among anti‐NID2 nanobodies, as the large molecular weight of NID2 limits accurate binding analysis by HPLC. All BLI measurements were performed on an Octet Red96e system using streptavidin (SA) biosensors (cat. no. 18‐5019). Nanobodies recognizing different NID2 domains were grouped for competitive binding analysis. BLI experiments were performed using the Octet buffer described above (20 mmol·L^−1^ HEPES, 150 mmol·L^−1^ NaCl, 0.2% BSA, pH 7.4), at 22 °C.

The assay workflow consisted of the following steps: Baseline, SA biosensors were equilibrated in Octet buffer for 60 s. Loading, sensors were transferred into Octet buffer containing biotinylated NID2‐FL‐Avi (62.79 nmol·L^−1^) and incubated for 100 s to immobilize the antigen. Wash, sensors were rinsed for 60 s. Association 1, antigen‐loaded sensors were incubated with the first nanobody (125 nmol·L^−1^) for 100 s. Association 2 (competition), sensors were subsequently transferred to a solution containing the second nanobody (125 nmol·L^−1^) and incubated for 100 s.

Group 1 comprised six distinct nanobody competition pairs, whereas Group 2 comprised four distinct competition pairs. BLI sensorgrams were visualized and compared using graphpad prism 9.5. Epitope competition was assessed qualitatively based on changes in binding response during the second association step, where a marked reduction or absence of additional signal indicated competitive binding, whereas a sustained binding response indicated non‐competitive binding.

#### Affinity determination of NID2 nanobodies

The binding affinities of nanobodies recognizing distinct epitopes within the G1G2 and rod–G3 domains of NID2 were determined using BLI on an Octet Red96e instrument with streptavidin (SA) biosensors (cat. no. 18‐5019). BLI experiments were performed using the Octet buffer described above (20 mmol·L^−1^ HEPES, 150 mmol·L^−1^ NaCl, 0.2% BSA, pH 7.4), at 22 °C. Biotinylated NID2‐FL‐Avi protein was immobilized onto SA biosensors prior to kinetic analysis. NID2‐FL‐Avi was serially diluted from 1000 to 15.625 nmol·L^−1^.

The assay workflow consisted of the following steps: Baseline, SA biosensors were equilibrated for 150 s. Loading, sensors were transferred to biotinylated NID2‐FL‐Avi and incubated for 150 s to immobilize the antigen. Wash, sensors were rinsed for 150 s. Association, antigen‐loaded sensors were immersed in nanobody solutions for 150 s. Dissociation, sensors were subsequently transferred back into buffer for 150 s to monitor dissociation.

Sensorgrams were analyzed using Octet Data Analysis software. Kinetic parameters, including the association rate constant (*k*_on_), dissociation rate constant (*k*_off_), and equilibrium dissociation constant (KD), were obtained by global fitting of the data to a 1 : 1 Langmuir binding model. For each nanobody, values are reported as mean ± standard deviation derived from multiple concentration curves.

### Development of a sandwich ELISA for NID2 detection based on dual nanobodies

Unless otherwise stated, all ELISA procedures used for the development and evaluation of the nanobody‐based sandwich ELISA were performed using detergent‐free buffer systems. PBS (pH 7.4) supplemented with 2% (w/v) BSA was used as the blocking buffer, whereas PBS supplemented with 0.2% (w/v) BSA was used as the wash buffer as well as for sample and antibody dilution. Unless otherwise stated, all ELISA incubations were performed at 22 °C on a horizontal shaker under gentle agitation.

#### Verification of nanobody binding intensity by ELISA

To evaluate the binding of individual nanobodies to NID2, a direct ELISA format was employed. ELISA plates were coated with full‐length NID2 (NID2‐FL) as the immobilized antigen. Candidate nanobodies (NB‐1C3, NB‐2E6, and NB‐1E5) were serially diluted and incubated with the coated antigen for 1 h at 22 °C with shaking at 200 rpm. After washing, bound nanobodies were detected using an HRP‐conjugated mouse anti‐HA antibody incubated for 30 min under the same conditions. Signal development was achieved using 3,3′,5,5′‐tetramethylbenzidine (TMB) substrate, and the reaction was terminated by acid addition. Absorbance at 450 nm was measured using a microplate reader. Binding intensities were compared based on OD450 values, and nanobodies exhibiting strong and concentration‐dependent binding signals were selected for subsequent sandwich ELISA development.

#### Construction, expression, purification, and Biotinylation of NB‐2E6‐Avi

Based on the screening results, NB‐2E6 was selected as the capture nanobody for sandwich ELISA development. An Avi tag was introduced at the C terminus of NB‐2E6 using BamHI and HindIII restriction sites, and the resulting construct was cloned into the pET28a vector to generate the pET28a‐NB‐2E6‐Avi plasmid.

The recombinant plasmid was transformed into BL21 competent cells for expression. Purification of NB‐2E6‐Avi was performed following the same procedure described for other nanobodies. The molecular weight and purity of NB‐2E6‐Avi were verified by SDS/PAGE.

*In vitro* biotinylation of NB‐2E6‐Avi was carried out using a BirA‐based enzymatic biotinylation system at 30 °C for 40 min, with reaction components and proportions summarized in Table 5. Following the biotinylation reaction, the resulting NB‐2E6‐Avi‐biotin was used for downstream applications. Successful biotinylation of Avi‐tagged nanobodies was verified by streptavidin–HRP blotting. Briefly, protein samples were separated by SDS/PAGE and transferred onto PVDF membranes. After blocking, membranes were incubated with HRP‐conjugated streptavidin to specifically detect biotinylated proteins. Signal detection was performed using an enhanced chemiluminescence substrate according to the manufacturer's instructions.

**Table 5:** Biotinylation system for NB‐2E6‐Avi.

Reagent	Volume (μL)
BirA	375
NB‐2E6‐Avi(3 mg·mL^−1^)	3000
10× Biotin‐L‐Buffer‐A	422
10× Biotin‐L‐Buffer‐B	422
Total	4219

#### Establishment of dual nanobody sandwich ELISA for NID2 detection

NeutrAvidin was added to each well of a 96‐well microplate at a concentration of 5 μg·mL^−1^ (100 μL per well). Plates were sealed and incubated overnight (12 h) at 4 °C on a horizontal shaker at 100 rpm. The following day, the NeutrAvidin solution was discarded by inversion, and plates were washed three times with wash buffer.

Plates were blocked with 100 μL of blocking buffer per well and incubated for 1 h. After washing, biotinylated NB‐2E6‐Avi (5 μg·mL^−1^) was added to the experimental and negative control wells and incubated for 1 h. Plates were washed, followed by the addition of 100 μL of antigen samples diluted in wash buffer and incubation for 1 h.

After washing, NB‐1C3 (1 μg·mL^−1^) was added to each well and incubated for 1 h. Plates were washed, and 100 μL of HRP‐conjugated anti‐HA mouse monoclonal antibody (diluted 1 : 2000) was added and incubated for 30 min. After final washing, 100 μL of TMB substrate solution was added to each well and allowed to react for 3 min in the dark. The reaction was terminated by adding 100 μL of 2 m H_2_SO_4_, and absorbance was measured at 450 nm using a microplate reader.

## Results

### Purification of NID2‐FL, NID2‐FL‐Avi, and NID2‐G1G2 proteins

PCR amplification of the NID‐FL‐Avi and NID2‐G1G2 fragments was performed, and the products were verified by 1% agarose gel electrophoresis (Fig. S1A). After ligation and transformation, colony PCR was used to confirm the successful insertion of the target fragments into the vectors (Fig. S1B). Clones with correct insertions were sent to Suzhou GenScript Biotech Co., Ltd., for sequencing and subsequently returned.

**Fig. 2:**
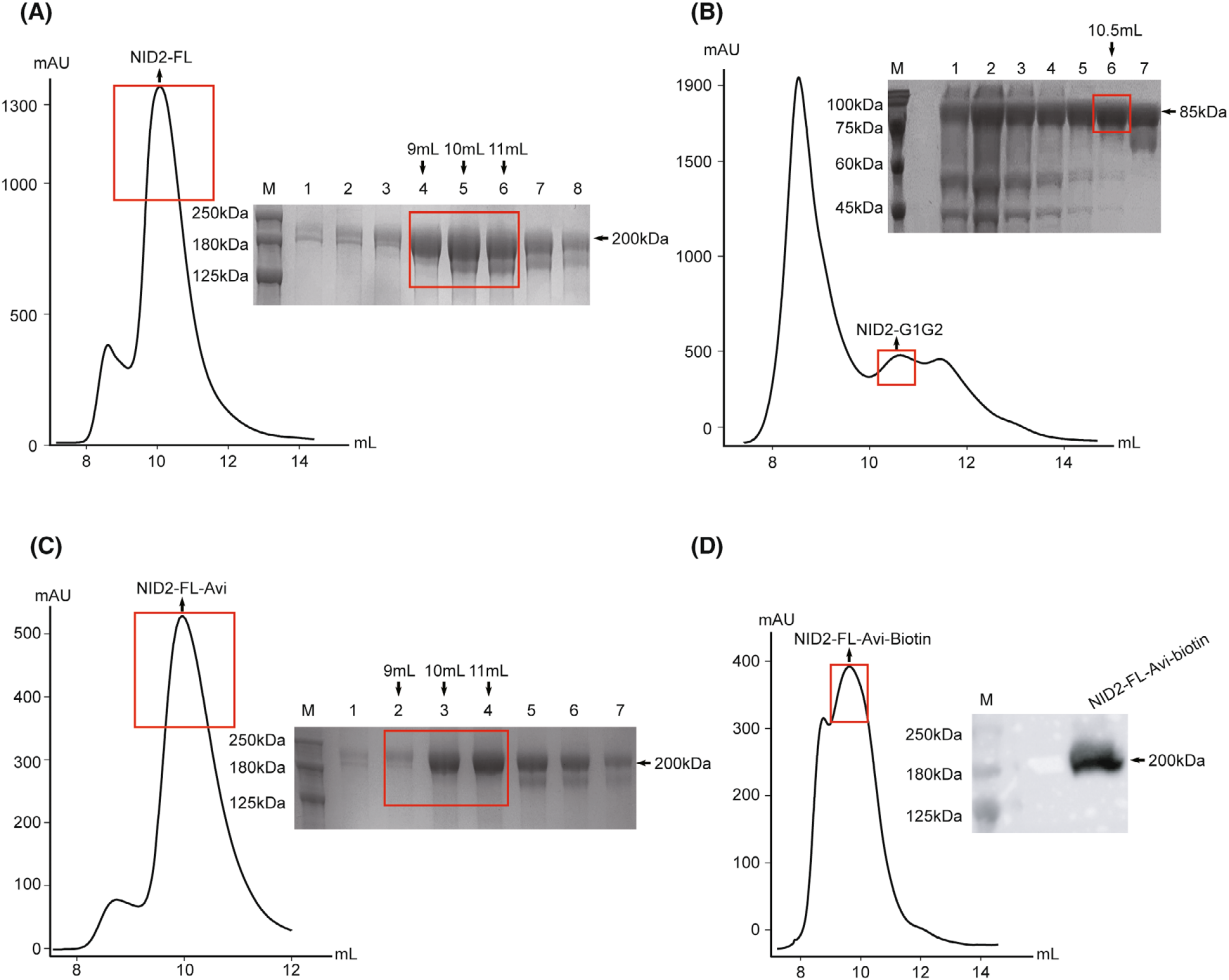
Expression, purification, and biotinylation of recombinant NID2 proteins. (A) SEC profile of purified NID2‐FL‐Avi on a Superdex™ 200 Increase 10/300 GL column. The main peak eluted at approximately 9–11 mL. Fractions corresponding to the main peak were analyzed by SDS/PAGE. (B) SEC profile of biotinylated NID2‐FL‐Avi‐biotin on a Superdex™ 200 Increase 10/300 GL column. Biotinylation was confirmed by streptavidin–HRP blotting. (C) SEC purification of NID2‐FL on a Superdex™ 200 Increase 10/300 GL column and SDS/PAGE analysis of peak fractions. (D) SEC purification of NID2‐G1G2 on a Superdex™ 200 Increase 10/300 GL column. The main peak eluted at approximately 10.5 mL, and corresponding fractions were analyzed by SDS/PAGE.

The purification results of NID2‐FL (Fig. 2A), NID2‐G1G2 (Fig. 2B), and NID2‐FL‐Avi (Fig. 2C) proteins are shown. The theoretical molecular weight of NID2‐FL is approximately 150 kDa; however, likely due to PTMs during expression, the SDS/PAGE bands appeared around 200 kDa. In contrast, NID2‐G1G2 migrated at the expected molecular weight of ~ 85 kDa on SDS/PAGE. Streptavidin–HRP blotting analysis revealed a clear band of NID2‐FL‐Avi‐biotin at 200 kDa (Fig. 2D), confirming the presence of biotinylated NID2‐FL‐Avi protein and its suitability for subsequent nanobody screening.

### Construction of an anti‐NID2 nanobody library

**Fig. 3:**
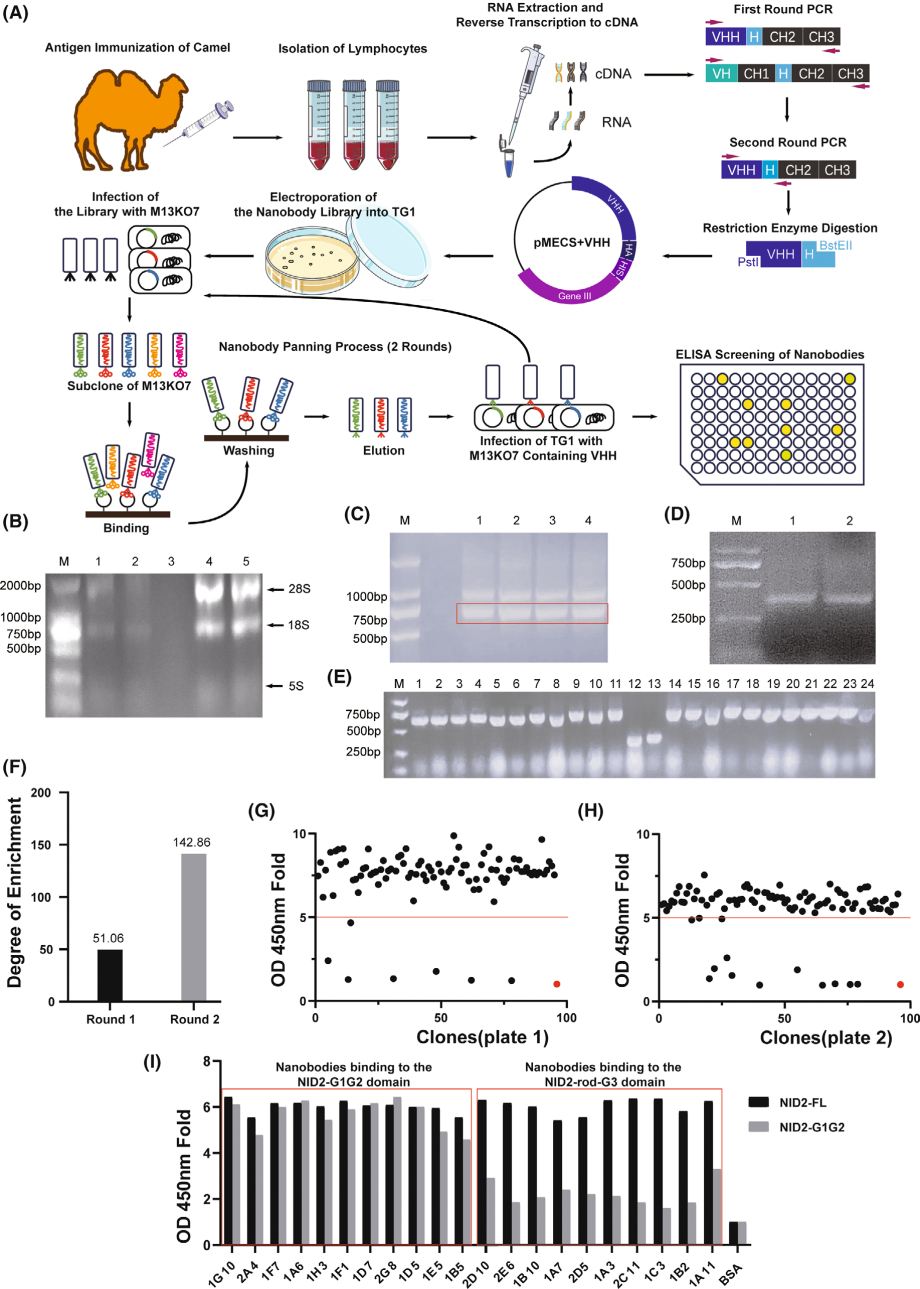
Screening process of anti‐NID2 nanobodies. (A) Flowchart of nanobody library construction and nanobody screening using phage display technology. (B) Agarose gel electrophoresis of total RNA extracted from camel peripheral blood. M, 2000 DNA marker. (C) The first round of nested PCR yielded nanobody‐containing gene fragments at 750 bp. (D) The second round of nested PCR, with gene fragments from the first round as templates, yielded nanobody‐containing gene fragments at 400 bp. (E) Verification of the positive insertion rate of nanobody fragments into PMECS phagemids. (F) Two rounds of panning for anti‐NID2 nanobodies using phage screening technology. (G, H) Reading of ELISA results using a microplate reader, where red dots represent the blank control group. (I) Differentiation of nanobodies binding to by ELISA. Data are presented as mean ± SD of three independent biological replicates.

As shown in the nanobody screening process (Fig. 3A), total RNA was extracted from the peripheral blood of post‐immunization camels (Fig. 3B). RNAs corresponding to Lanes 4 and 5 were selected for reverse transcription into cDNA. After the first round of nested PCR, 2% agarose gel electrophoresis confirmed bands at 1000 and 750 bp (Fig. 3C). The band at 750 bp was subjected to gel extraction. Using this gel‐extracted product from the first round of nested PCR as a template, the second round of nanobody fragment amplification was performed, yielding a 400‐bp fragment (Fig. 3D). The amplified nanobody fragments were cloned into the pMECS vector, and the plasmids were transformed into TG1 competent cells using an electroporator. The positive insertion rate of nanobodies was determined to be 91.6% by colony PCR (Fig. 3E), thus completing the construction of the anti‐NID2‐FL library, with a determined library capacity of 1.46 × 10^10^ CFU·mL^−1^.

### Immunopanning and identification of anti‐NID2 Nanobodies

The constructed library was subjected to two rounds of panning, ultimately obtaining an antibody library with an enrichment degree of 142.56 (Fig. 3F). The panned antibody library was appropriately diluted and plated on 2YT‐AMP‐GLU plates. Colonies were picked and nanobody expression plates were prepared. The binding activity of nanobodies to NID2‐FL was determined by ELISA (Fig. 3G,H), and positive clones with an OD450 value exceeding five times that of the blank control were screened out.

The corresponding positive nanobodies were subjected to large‐scale expression and purification (Fig. S2) and recharacterized by ELISA with NID2‐FL and NID2‐G1G2. The microplate reader readings were compared, and the average reading of each group (including the blank group) was calculated, with the average value of the blank group set as the reference value of 1. The average values of the two experimental groups were compared proportionally, and after proportional calculation of the experimental group readings, the following criteria were adopted: When the reading of a nanobody on NID2‐G1G2 was less than 55% of that on NID2‐FL (i.e., ratio < 0.55), it was determined to primarily bind to NID‐rod‐G3. When a nanobody showed high readings on both NID2‐FL and NID2‐G1G2, with a ratio ≥ 0.75, it was determined to primarily bind to the G1‐G2 domain. Total 21 nanobodies were divided into 11 nanobodies binding to NID2‐G1G2 and 10 nanobodies binding to NID2‐rod‐G3 (Fig. 3I).

### Competition assay of nanobodies against different domains of NID2

To further resolve the epitope relationships among anti‐NID2 nanobodies, BLI‐based competition assays were performed using sequential nanobody binding to immobilized NID2‐FL‐Avi. This assay monitored two successive interactions: initial binding of NID2 to the first nanobody, followed by evaluation of whether a second nanobody could further associate with the antigen.

When two nanobodies recognized distinct, non‐overlapping epitopes, binding of the first nanobody produced an initial increase in response that reached a steady state, and subsequent introduction of the second nanobody resulted in a further increase in signal, generating a second ascending phase. In contrast, when two nanobodies competed for the same or overlapping epitopes, no additional increase in response was observed during the second association step, and the signal remained at a plateau or showed only minimal fluctuations.

**Fig. 4:**
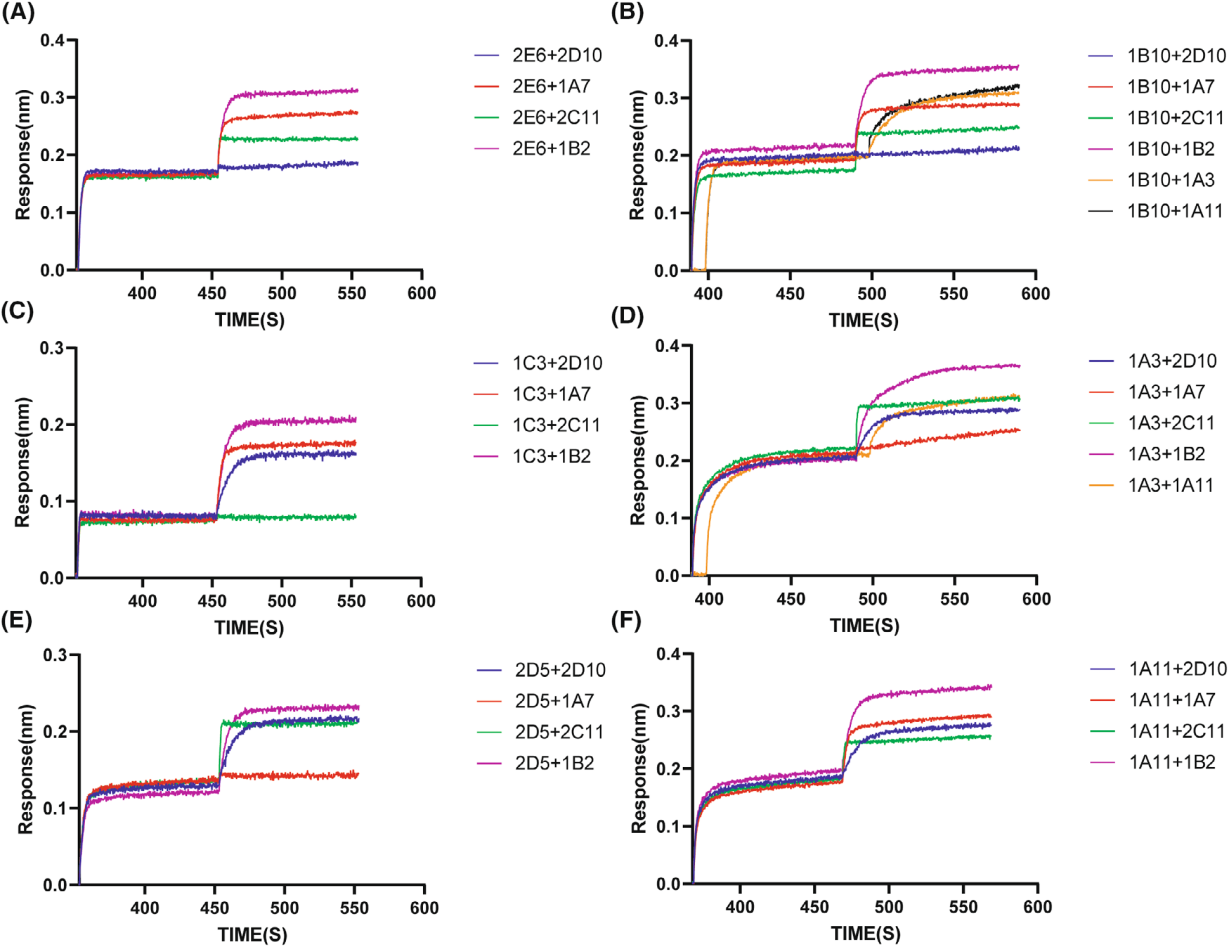
Epitope competition analysis of anti‐NID2 nanobodies targeting the rod–G3 domain by BLI. (A–F) Representative BLI competition sensorgrams showing sequential binding of rod–G3–targeting nanobodies to immobilized NID2‐FL‐Avi. Based on the competition patterns observed in the second association step, the nanobodies were classified into five distinct, non‐overlapping epitope groups within the rod–G3 domain. Panels (A–F) show representative data from a single experiment (no biological replicates).

**Fig. 5:**
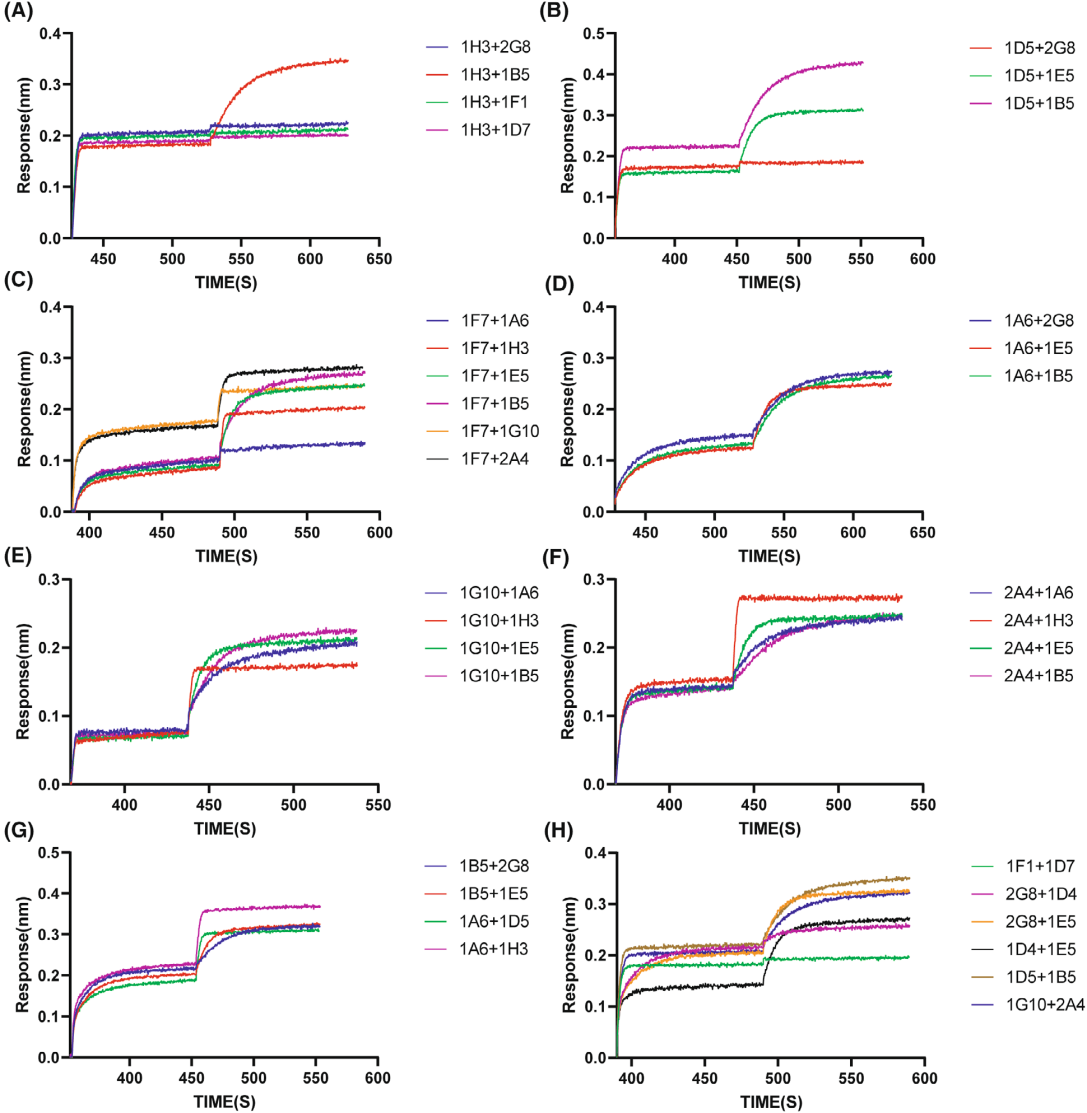
Epitope competition analysis of anti‐NID2 nanobodies targeting the G1G2 domain by BLI. (A–H) Representative BLI competition sensorgrams showing sequential binding of G1G2‐targeting nanobodies to immobilized NID2‐FL‐Avi. Analysis of the competition profiles allowed classification of the nanobodies into six distinct, non‐overlapping epitope groups within the G1G2 domain. Panels (A–H) show representative data from a single experiment (no biological replicates).

Representative competition sensorgrams are shown in Figs 4A–F, 5A–H. Based on these qualitative binding patterns, nanobodies exhibiting epitope competition were classified as recognizing the same epitope, whereas nanobodies that did not compete were assigned to distinct epitope groups. Using this epitope binning strategy, multiple nonoverlapping epitopes were identified within both the G1G2 and rod–G3 domains of NID2. The complete epitope grouping and domain assignments of the nanobodies are summarized in Table 6.

**Table 6:** Identification of non‐epitope competing nanobodies targeting NID2 G1G2 and Rod‐G3 domains.

Binding epitope cluster	1	2	3	4	5	6
Rod‐G3 domain	**2E6**, 2D10, 1B10	**1C3**, 2C11	1A7, 2D5, 1A3	1B2	1A11	
G1G2 domain	1H3, 1F1, 1D7, 2G8, 1D5	1F7,1A6	1G10	2A4	1E5	1B5

Bold values indicate the two nanobodies selected for sandwich ELISA, which also showed the highest binding affinity in our experiments.

### Affinity determination of anti‐NID2 nanobodies

The binding kinetics of anti‐NID2 nanobodies were analyzed using the Octet Red96e system to determine the association rate constant (*k*_on_), dissociation rate constant (*k*_off_), and equilibrium dissociation constant (KD). Serial dilutions of NID2‐FL‐Avi (1000–15.625 nm) were used to evaluate nanobody–antigen interactions during the association and dissociation phases. Kinetic parameters were obtained by global fitting of the BLI sensorgrams using the Octet Data Analysis software.

**Fig. 6:**
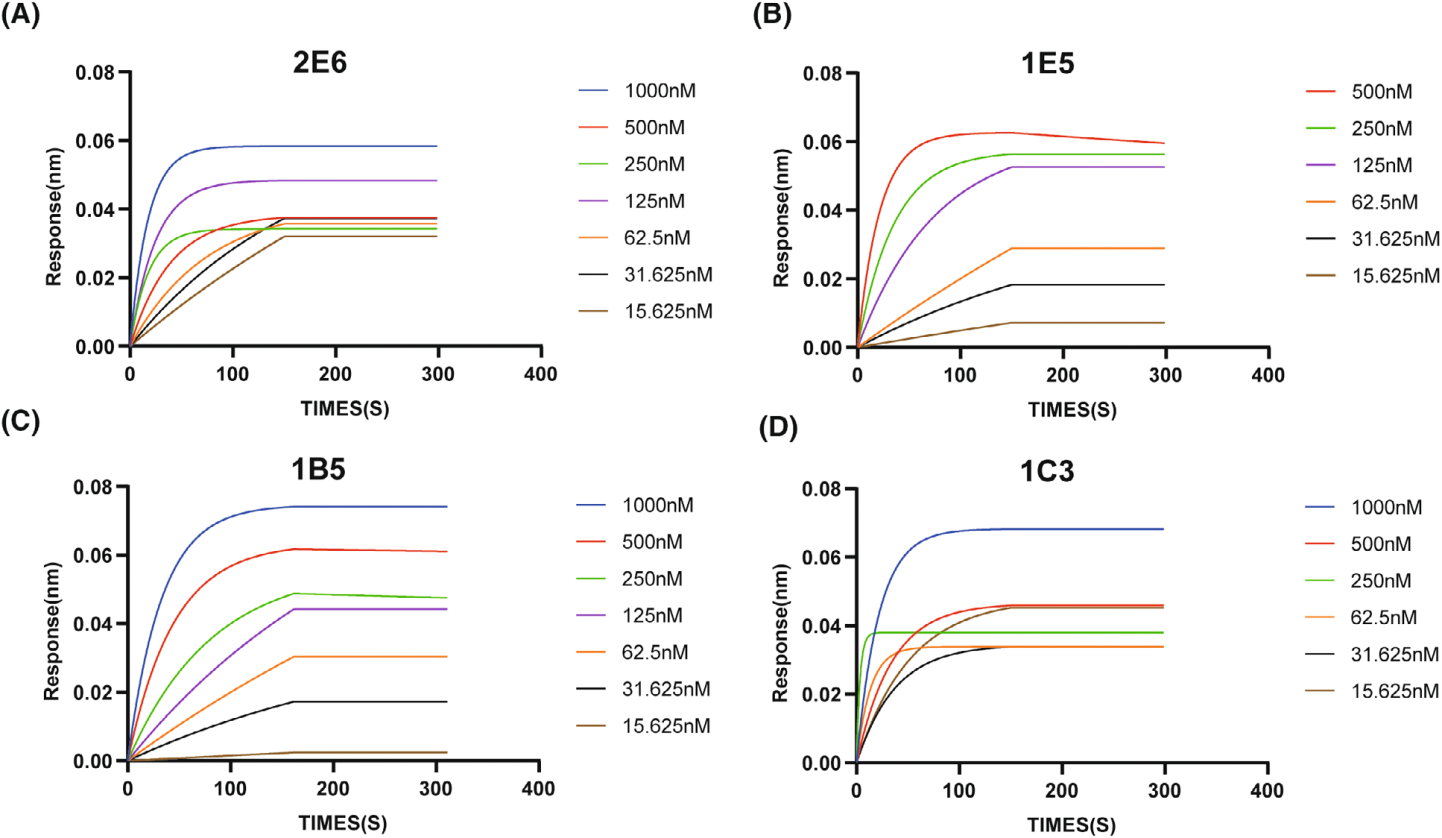
Affinity Determination Results of Anti‐NID2‐FL Nanobodies. (A) Representative binding and dissociation sensorgrams of NB‐2E6 interacting with immobilized NID2‐FL‐Avi at the indicated concentrations. (B) Representative binding and dissociation sensorgrams of NB‐1E5 interacting with immobilized NID2‐FL‐Avi at the indicated concentrations. (C) Representative binding and dissociation sensorgrams of NB‐1B5 interacting with immobilized NID2‐FL‐Avi at the indicated concentrations. (D) Representative binding and dissociation sensorgrams of NB‐1C3 interacting with immobilized NID2‐FL‐Avi at the indicated concentrations. For NB‐2E6 and NB‐1C3, dissociation rates reached the lower detection limit of the Octet system; therefore, the reported KD values represent apparent affinities constrained by instrumental sensitivity. Each concentration was measured once; panels show representative sensorgrams from these single measurements (no biological replicates).

Representative binding sensorgrams for nanobodies NB‐2E6, NB‐1E5, NB‐1B5, and NB‐1C3 are shown in Fig. 6A–D, respectively. As summarized in Table 7, nanobodies NB‐2E6 (Fig. 6A) and NB‐1C3 (Fig. 6D) exhibited extremely slow dissociation kinetics, with k_off_ values reaching the lower detection limit of the instrument (1.00 × 10^−7^ s^−1^). Consequently, the calculated KD values for these nanobodies were in the picomolar range and constrained by the analytical sensitivity of the system.

**Table 7:** Summary of kinetic parameters of anti‐NID2 nanobodies. Kinetic parameters were obtained by global fitting of the BLI sensorgrams using Octet Data Analysis software. Each concentration was measured once. The reported errors reflect fitting uncertainty across the concentration series rather than replicate‐to‐replicate variation.

Parameter	NB‐2E6	NB‐1E5	NB‐1B5	NB‐1C3
*k*_on_ (M^−1^·s^−1^)	(1.74 ± 0.97) × 10^5^	(8.90 ± 3.57) × 10^4^	(4.81 ± 3.12) × 10^4^	(7.66 ± 5.70) × 10^5^
*k*_off_ (s^−1^)	≤ 1.00 × 10^−7^	(5.64 ± 13.79) × 10^−5^	(3.50 ± 6.64) × 10^−5^	≤ 1.00 × 10^−7^
KD (M)	≤ 1.00 × 10^−12^	(6.11 ± 14.94) × 10^−10^	(7.39 ± 13.86) × 10^−10^	≤ 1.00 × 10^−12^
*n* (concentrations)	7	6	7	6

In contrast, nanobodies NB‐1E5 (Fig. 6B) and NB‐1B5 (Fig. 6C) displayed faster dissociation rates, resulting in equilibrium affinities in the nanomolar range. These results demonstrate distinct kinetic behaviors among nanobodies recognizing different epitopes of NID2.

### Development of a Sandwich enzyme‐linked immunosorbent assay for NID2 detection based on dual nanobodies

**Fig. 7:**
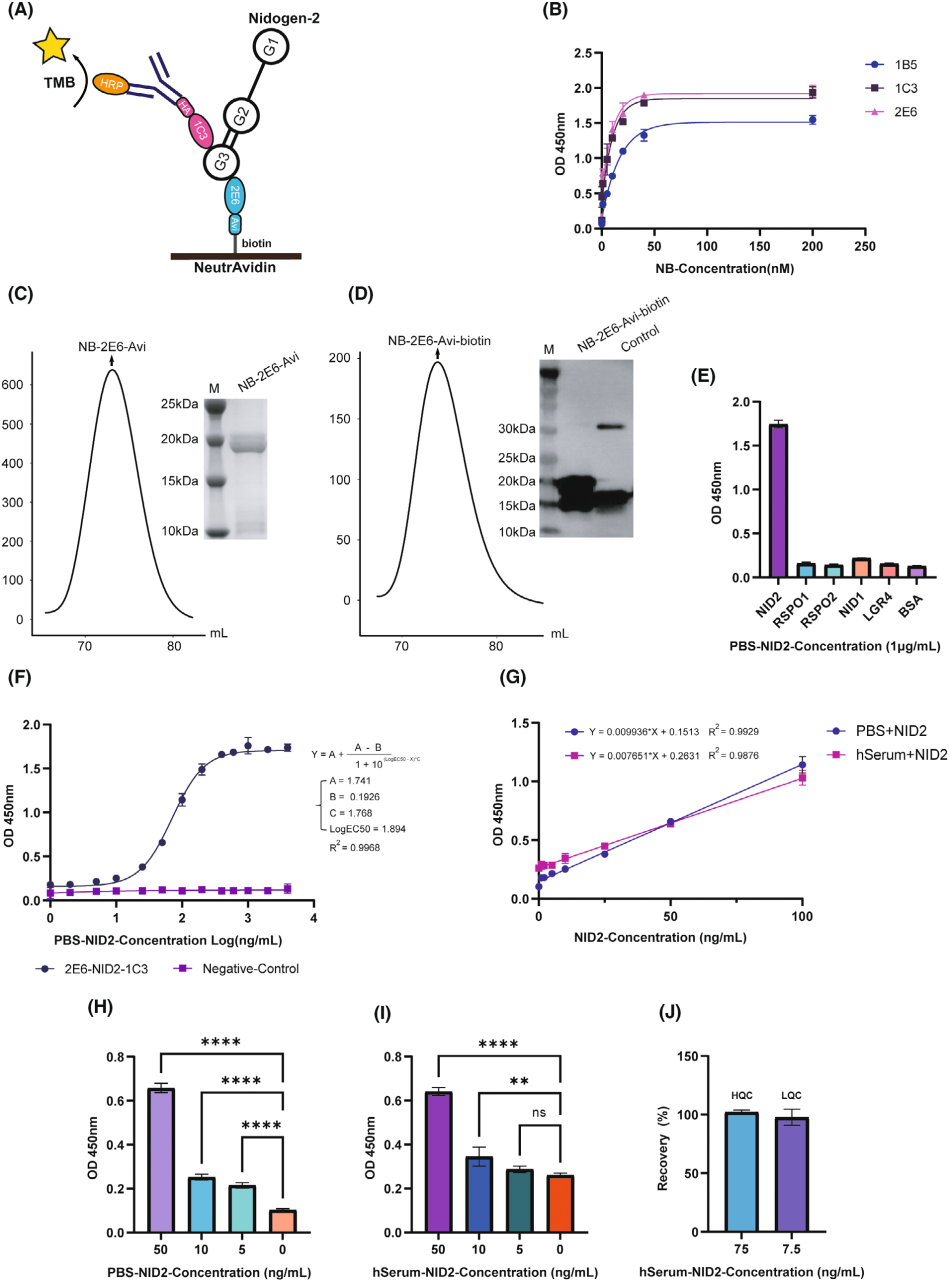
Construction of a Sandwich Enzyme‐Linked Immunosorbent Assay for NID2 Detection Using Dual Nanobodies. (A) Schematic diagram of the dual‐nanobody sandwich ELISA structure for NID2 detection. (B) Affinity verification of NB‐1E5, NB‐1C3, and NB‐2E6 nanobodies via ELISA. (C, D) Size exclusion chromatography, 15%‐SDS/PAGE, and western blot results for biotinylation verification of NB‐2E6‐Avi. (E) Cross‐reactivity assay of the dual‐nanobody sandwich ELISA. (F) Detection results of NID2 concentration gradients (4 to 1 μg·mL^−1^) diluted using PBS as the matrix. (G) Standard curves of NID2 (0 to 100 μg·mL^−1^) diluted using PBS and healthy human serum as matrices. (H) Statistical analysis of significant differences in NID2 at different concentrations diluted using PBS as the matrix. (I) Statistical analysis of significant differences in NID2 at different concentrations diluted using healthy human serum as the matrix. (J) Determination of recovery rates for HQC and LQC detected values using healthy human serum as the matrix. Data are presented as mean ± SD from *n* = 3 independent biological replicates. Statistical significance was determined by one‐way ANOVA followed by Tukey's multiple comparisons test. *P* values are indicated as follows: *P* < 0.05 (*), *P* < 0.01 (**), *P* < 0.001 (***), *P* < 0.0001 (****); ns, not significant.

The top three nanobodies with the highest affinity from the aforementioned experiments—NB‐1E5, NB‐1C3, and NB‐2E6—were selected for verification of binding strength via ELISA. A series of eight concentrations of nanobodies were set: 200, 40, 20, 10, 5, 1, 0.2, and 0.04 nm (Fig. 7A). After four‐parameter fitting of the OD450 readings, it was evident that the binding capacity of NB‐1E5 was significantly weaker than that of NB‐1C3 and NB‐2E6, while the binding capacity of NB‐2E6 was slightly higher than that of NB‐1C3 (Fig. 7A). Therefore, for the dual‐nanobody sandwich ELISA, the highest‐affinity NB‐2E6 was chosen as the capture antibody, and NB‐1C3 as the detection antibody. Since the PMECS vector inherently contains a C‐terminal HA tag, NB‐1C3 could be specifically recognized by an HRP‐conjugated mouse anti‐HA antibody, enabling selective detection of antigen‐bound NB‐1C3 in solution (Fig. 7B). Importantly, the capture nanobody NB‐2E6‐Avi was expressed from a pET28a‐based construct and does not contain an HA tag, thereby preventing direct recognition by the anti‐HA antibody and minimizing background signal.

The elution peak of NB‐2E6‐Avi after purification using a HiLoad™ Superdex 75 pg Increase column was normal. The predicted molecular weight of NB‐2E6‐Avi expressed in the pET28a vector was approximately 19 kDa (Fig. 7C). The purified NB‐2E6‐Avi was biotinylated and then re‐purified using the HiLoad™ Superdex 75 pg Increase column. Western blot verification showed that the BirA enzyme (around 30 kDa) was almost completely removed, and a distinct band was observed around 20 kDa (Fig. 7D). Thus, NB‐2E6‐Avi‐biotin was suitable for subsequent experiments.

A cross‐reactivity assay was performed on this pair of nanobodies to ensure their specific binding to NID2 (Fig. 7E). NeutrAvidin was coated onto the microplate and incubated with biotinylated NB‐2E6‐Avi at a 1 : 1 molar ratio to allow high‐affinity, noncovalent binding, thereby generating a stable solid phase for capturing the target antigen, followed by incubation with 1 μg·mL^−1^ of antigen proteins: NID2, RSPO1 (R‐spondin 1), RSPO2 (R‐spondin 2), NID1 (Nidogen‐1), and LGR4 (leucine‐rich repeat containing G protein‐coupled receptor 4). Subsequently, NB‐1C3 (with a C‐terminal HA tag) was used to capture the target antigen. Color development was achieved by incubating with HRP‐conjugated mouse anti‐HA antibody followed by TMB reaction. The OD450 value of NID2 was significantly higher than that of other antigens, confirming that the sandwich nanobody pair (NB‐2E6 and NB‐1C3) enables specific detection of NID2.

NID2‐FL was set at 14 concentration gradients: 4, 2^1^, 1 μg mL^−1^, 600, 400, 200, 100, 50, 25, 10, 5, 2, 1, and 0 ng·mL^−1^. A negative control group was also included. IL‐36R, another common serum protein, was diluted in PBS at the same gradients and measured using the same sandwich method under serum‐free conditions. The OD450 readings were subjected to logarithmic transformation, and 13 gradients (from 1 ng·mL^−1^ to 4 μg·mL^−1^) were used for four‐parameter fitting (Fig. 7F). The *R* ^2^ value was > 0.99, indicating high reliability of the curve, and the readings of the negative control group were as expected compared to the experimental groups.

A series of seven low concentration points (100, 50, 25, 10, 5, 2, and 1 ng·mL^−1^) was prepared by dilution into healthy human serum. Additionally, two concentrations (75 and 7.5 ng·mL^−1^) were included as low‐quality control (LQC) and high‐quality control (HQC) samples to verify recovery rates. Comparison of the standard curves with and without serum (Fig. 7G) showed that in serum‐containing samples, nonspecific adsorption in serum interfered with the results at extremely low concentrations. Moreover, above 50 ng·mL^−1^, certain components in serum affected binding efficiency, resulting in slightly lower readings than the serum‐free standard curve. Statistical analysis of significant differences (Fig. 7H,I) revealed no significant difference between serum‐diluted NID2 at 5 ng·mL^−1^ and the serum control without NID2, but significant differences gradually emerged when the concentration reached 10 ng·mL^−1^. For the serum‐containing standard curve, recovery rates of LQC (7.5 ng·mL^−1^) and HQC (75 ng·mL^−1^) were both within 95–105% (Fig. 7J). Collectively, these results demonstrate that this sandwich ELISA can detect NID2 in human serum at concentrations > 10 ng·mL^−1^, with reliable performance meeting standard requirements.

## Discussion

The ECM plays an essential role in cancer invasion and metastasis by regulating host matrix responses, and the ability of tumor cells to breach the surrounding ECM barrier is considered a key event in cancer progression [16, 17, 18]. As a specialized ECM structure, the basement membrane (BM) separates tissues, provides structural support, and regulates cell behavior and signal transduction [19]. As a core BM component, NID2 contributes to ECM stability through its G3 domain and the rod‐like region connecting G2 and G3 and has been implicated in tumor‐associated microenvironmental remodeling [1, 4]. Clinical studies have reported altered NID2 expression in multiple malignancies, including gastric cancer and hepatocellular carcinoma, highlighting its potential relevance as a disease‐associated marker [2, 5, 20, 21, 22].

In addition to its structural functions, the G1G2 domain of NID2 has been reported to preferentially activate the Gαq–PKCα signaling pathway and to participate in vascular calcification [3, 23]. Although the mechanistic links between NID2 and this signaling axis remain incompletely understood, accumulating evidence suggests that Gαq–PKCα signaling plays important roles in cardiovascular regulation, smooth muscle contraction, and tumor progression [24]. These findings underscore the biological relevance of domain‐specific NID2 interactions and support the development of domain‐targeted molecular tools.

To further characterize the functional properties of the selected nanobodies, binding kinetics were analyzed using BLI. Marked differences in association and dissociation behaviors were observed among nanobodies recognizing distinct NID2 epitopes. Notably, NB‐2E6 and NB‐1C3 exhibited picomolar‐range apparent affinities dominated by extremely slow dissociation rates, indicative of highly stable antigen–nanobody complexes. In contrast, nanobodies with moderate affinities displayed faster dissociation kinetics and greater variability in kinetic parameters, potentially reflecting conformational heterogeneity of NID2 or differential epitope accessibility. The exceptionally slow dissociation of NB‐2E6 and NB‐1C3 is advantageous for applications requiring sustained antigen capture, such as sandwich immunoassays, providing a strong rationale for their selection as a paired detection system.

In this study, six noncompeting nanobodies recognizing distinct epitopes within the G1G2 domain and five nanobodies targeting the rod–G3 domain of NID2 were identified. Among them, NB‐2E6 and NB‐1C3 were used to establish a nanobody‐based sandwich ELISA, enabling detection of recombinant NID2 at concentrations as low as 10 ng·mL^−1^ in human serum. Although this sensitivity is lower than that reported for commercial antibody‐based ELISA kits (e.g., Abcam ab313906, ~ 49 pg·mL^−1^), the nanobody‐based format offers advantages in recombinant production, batch‐to‐batch consistency, and scalability.

Importantly, reported serum NID2 concentrations in patients with esophageal squamous cell carcinoma, ovarian cancer, and hepatocellular carcinoma generally fall within the ng·mL^−1^ range. For example, median serum NID2 levels of 24.4 ng·mL^−1^ have been reported in esophageal squamous cell carcinoma patients, with concentrations above 32.6 ng·mL^−1^ associated with increased mortality risk [2]. These values lie within the detection range of the present assay, supporting its potential applicability for high‐throughput serum NID2 quantification.

Although the predicted molecular weight of NID2 is approximately 150 kDa, full‐length recombinant NID2 migrated at ~ 200 kDa on SDS/PAGE, consistent with its identity as a heavily modified ECM glycoprotein. While annotated PTM sites are enriched within the G1–G2 region, electrophoretic mobility is influenced not only by the number of modification sites but also by PTM occupancy, glycan or glycosaminoglycan chain composition, and overall protein architecture. Accordingly, the isolated G1–G2 fragment migrated close to its predicted molecular weight (~ 85 kDa), suggesting that PTMs within this region exert a limited effect on SDS/PAGE mobility under the conditions used, whereas the full‐length multidomain protein exhibits nonideal electrophoretic behavior.

Several limitations should be acknowledged. First, a direct head‐to‐head comparison with commercial ELISA kits using identical standards and serum matrices could not be performed. Second, NID2 undergoes complex PTMs *in vivo* and may be subject to proteolytic processing or interactions with other ECM components. As nanobody selection and assay development were based on HEK‐293F‐expressed recombinant NID2, the modification profile may not fully recapitulate that of native circulating or tissue‐derived NID2, potentially influencing epitope accessibility and assay performance.

In conclusion, this study generated a panel of high‐affinity nanobodies targeting distinct functional domains of NID2 and established a nanobody‐based sandwich ELISA for NID2 detection. Beyond diagnostic applications, these nanobodies provide versatile molecular tools for future investigations into NID2‐related biological processes, including ECM remodeling, tumor invasion, tissue fibrosis, and vascular calcification.

## Conflict of interest

The authors declare no conflict of interest.

## Author contributions

JW, QC, and ZL conceived and designed the project and the experimental methodology. JW, SZ, WF, and YW performed the experiments. JW analyzed and interpreted the data. DZ, QH, and YL provided materials and access to instruments. JX acquired funding. JW wrote the paper. All authors reviewed and approved the final manuscript.

## Supporting information


**Fig. S1.** Construction of pCMV‐NID2‐FL‐Avi‐Flag and pCMV‐NID2‐G1G2‐Flag Plasmids.
**Fig. S2.** Expression and purification of anti‐NID2 nanobodies.

## Data Availability

All data supporting the findings of this study are included within the article and its Supporting Information. Raw experimental data from ELISA, biolayer interferometry (BLI), protein purification, and nanobody screening are available from the corresponding author upon reasonable request. There are no restrictions on the use of these data.
