# Supplementary material for: Screening and epitope characterization of Nidogen‐2‐specific nanobodies
Source: FEBS Open Bio. 2026 Mar 4;16(8):1459–76. doi: 10.1002/2211-5463.70219 (PMC13398667; doi:10.1002/2211-5463.70219)
Supplement: Supplementary file 1 — Fig. S1. Construction of pCMV‐NID2‐FL‐Avi‐Flag and pCMV‐NID2‐G1G2‐Flag Plasmids. Fig. S2. Expression and purification of anti‐NID2 nanobodies. [file FEB4-16-1459-s001.pdf]

Supporting Information

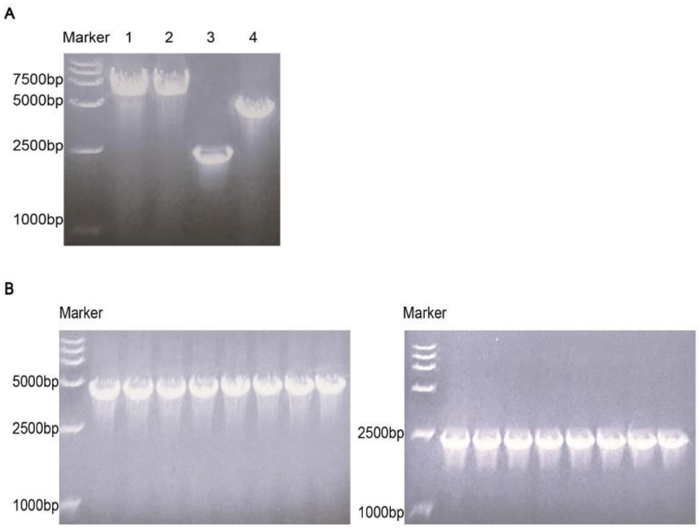

Figure S1. Construction of pCMV-NID2-FL-Avi-Flag and pCMV-NID2-G1G2-Flag Plasmids

(**A**) Lane 1 shows the PCR amplification product to the pCMV-Flag plasmid containing NID2-G1G2.Lane 2 shows the PCR amplification product to the pCMV-Flag plasmid containing NID2-FL-Avi.Lane 3 shows the PCR amplification product of NID2-G1G2.Lane 4 shows the PCR amplification product of NID2-FL-Avi. (**B**) PCR verification for the clones of NID2-FL-Avi(Left) and NID2-G1G2(Right).

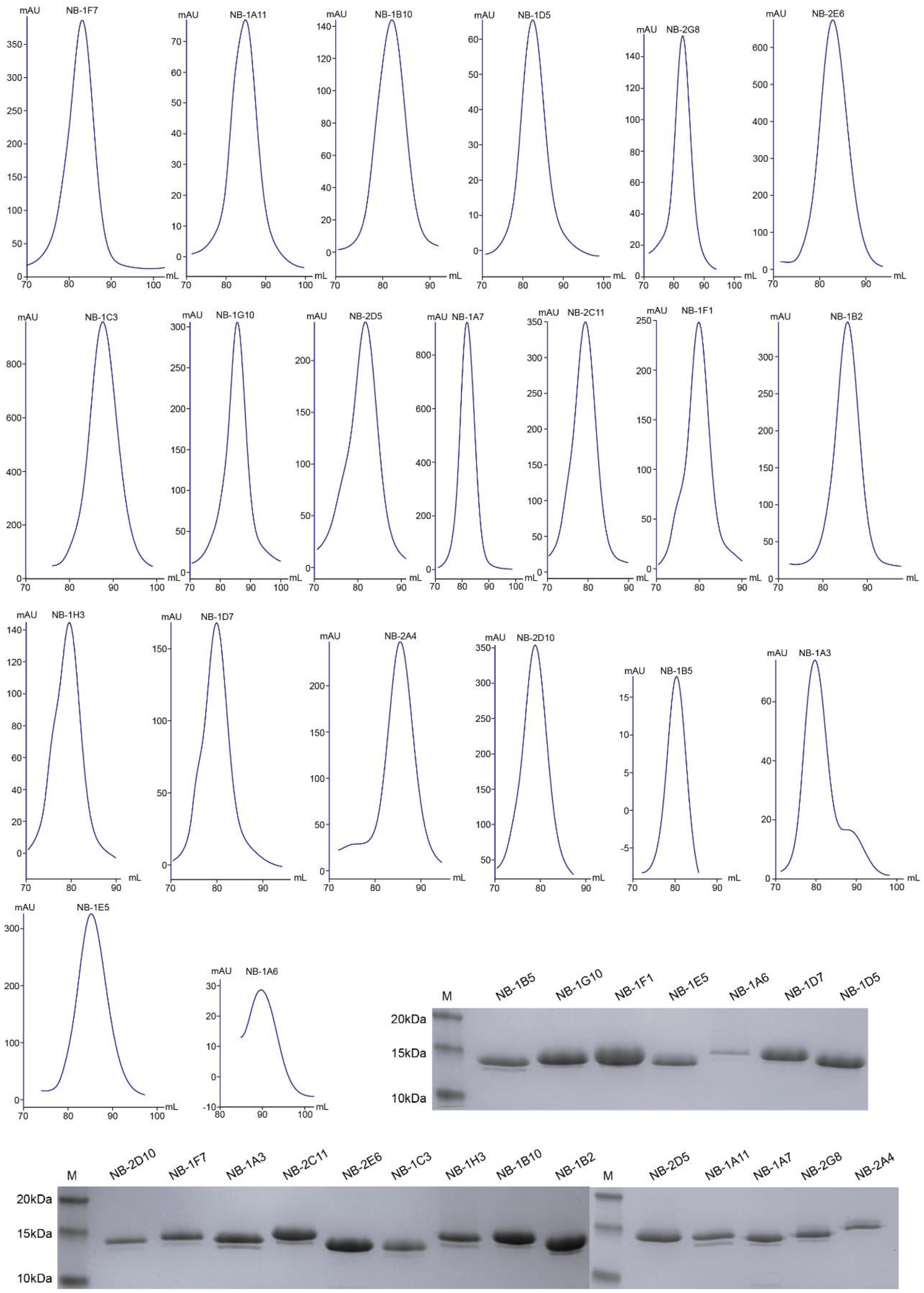

Figure S2. Expression and purification of anti-NID2 nanobodies.

Representative SDS–PAGE analysis of cytoplasmically expressed anti-NID2 nanobodies during purification. Nanobodies were expressed in Escherichia coli and purified by Ni–NTA affinity chromatography followed by SEC, yielding a predominant band at the expected molecular weight (~15 kDa).

Although periplasmic expression systems (e.g., pET-22b or pET-26b) can facilitate disulfide bond

formation and enable selective extraction of periplasmic proteins, cytoplasmic expression was employed in this study to maximize expression yield and streamline parallel purification of multiple nanobody clones. The observed monodispersity and purity of the nanobody preparations, as demonstrated by SDS–PAGE, indicate that cytoplasmic expression provided properly folded and functionally active nanobodies suitable for downstream biophysical and immunoassay applications.
